# Rapid Multi-Omics for Bacterial Identification Using Flow Injection–Ion Mobility–Mass Spectrometry

**DOI:** 10.1021/acs.analchem.5c00417

**Published:** 2025-06-24

**Authors:** Hannah M. Hynds, Jana M. Carpenter, Kelly M. Hines

**Affiliations:** Department of Chemistry, 1355 University of Georgia, Athens, Georgia 30602, United States

## Abstract

The implementation of mass spectrometry (MS) in clinical microbiology has made a significant improvement in the turnaround time from positive culture to identification, but current protein-based approaches can struggle with species-level identification because of the high degree of homology within a genus. However, other MS-based strategies for bacterial identification that are based on lipids and small molecules have shown promise toward species-level identification and detection of specific phenotypes, including those related to antibiotic resistance. We are leveraging rapid gas-phase ion mobility (IM) separations coupled to MS to simultaneously detect the lipids and metabolites in bacterial pathogens. Using flow-injection (FI) rather than liquid chromatography (LC), we instead rely more directly on the structural separation of the IM dimension to resolve features from different biochemical classes and aid in identification. A head-to-head comparison demonstrates that the FI-IM-MS multiomic strategy performs similarly to LC-IM-MS in its ability to distinguish 24 strains of the high-concern ESKAPE pathogens, while shortening overall analysis time from 17 to 2 min per injection. We demonstrate that the IM dimension has excellent stability and reproducibility, which enables extracted IM peak areas to be used in lieu of chromatographic peak areas. Furthermore, the same features that are important for the discrimination of bacterial species and strains are found within both the FI-IM-MS and HILIC-IM-MS data sets. These results showcase the capabilities of mobility-enabled rapid multiomics and open the possibility to detect subtle strain-level differences and resistance phenotypes in bacterial pathogens by including additional classes of biomolecules.

## Graphical abstract

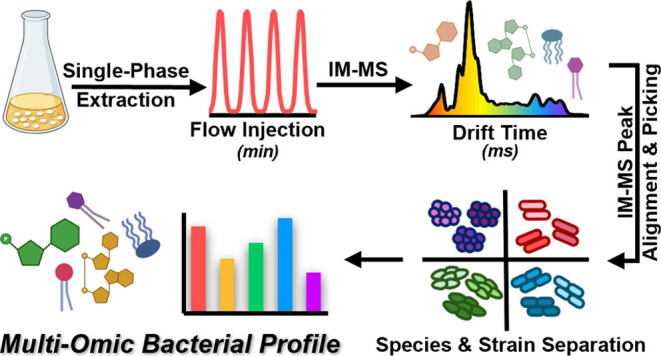

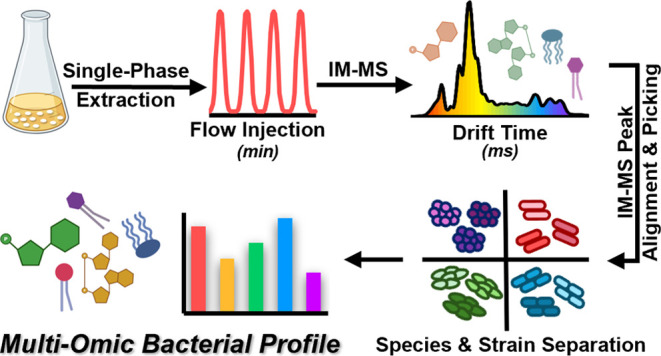

## Introduction

The investigation of a class of biomolecules and their corresponding biological processes, known as omics, has gained growing attention in the past decade due to its significant correlation with the causation and progression of disease states. While single-omic techniques like genomics,^1–3^ transcriptomics,^4^ and proteomics^5–7^ have provided comprehensive profiles of individual biomolecular groups, it has been demonstrated that the combination or integration of multiple omic techniques yields a more comprehensive understanding of the biological systems under investigation.^8,9^ A typical multiomics study breaks down biological samples into separate biochemical components, analyzes each type of biomolecule separately, and then integrates the resulting data sets using advanced bioinformatics strategies. While the knowledge gleaned from these experiments is exceptionally insightful, the time and resource requirements for the analytical and informatic pipelines typically limit the use of multiomics approaches to mechanistic or discovery-type studies.

One area that could benefit from rapid multiomic strategies is clinical microbiology, where mass spectrometry has become the standard method for the identification of unknown bacterial species. Platforms like the bioMérieux Vitek MS and Bruker Biotyper rely on protein fingerprint spectra that are generated by matrix-assisted laser desorption/ionization and time-of-flight mass spectrometry (MALDI-ToF-MS) to differentiate bacteria at the genus and species level.^10–15^ Other strategies that are in development target differences in lipids between bacterial species. MALDI-ToF-MS and similar approaches have been used to detect glycolipids,^16–19^ fatty acids,^20–22^ and phospholipids,^23,24^ while direct sampling strategies like the MasSpec Pen,^25^ paper spray,^26–28^ or rapid evaporative ionization MS (REIMS)^29–31^ rely mostly on phospholipids, fatty acids, and other lipophilic molecules. Other MS-based bacterial identification strategies incorporate liquid chromatography with MS to measure the signature metabolites that are produced and consumed by different bacteria.^32,33^ While each of these methods have shown success in identifying bacteria based on a subset of the organism’s biomolecular composition, it is reasonable to expect that gains in the specificity or sensitivity of bacterial identification could be improved by using a multiomics approach to detect differences across several classes of biomolecules. However, the implementation of multiomics at the speed and scale required for diagnostic settings like clinical microbiology requires a significant improvement in the throughput of the multiomics strategy.

An alternative strategy to the standard multiomics workflow would be to forego the initial partitioning of the biological sample and preserve its inherent “multiomic” composition from the start. From an analytical perspective, there are several reasons why this would be a difficult approach to implement—first among them being the incompatible chemical properties (i.e., solubility, polarity, etc.) of the biomolecules themselves. Other difficulties, such as ionization suppression and interferences, can be addressed to some extent with liquid chromatography, but solubility issues still limit the breadth of chemical complexity that is feasible in samples for chromatographic separations. On the other hand, gas-phase separations based on ion mobility (IM) provide some capability to resolve similar mass or isobaric analytes that differ in their structures. The combination of IM with MS (IM-MS) presents the additional advantage of biomolecular-specific trendlines that arise from differences in the relationships between size and mass across lipids, metabolites, peptides/proteins, etc.^34–36^ Although IM drift times vary between instruments, the calibration or normalization of drift times to collision cross sections (CCS) provides a stable molecular descriptor that can be shared across laboratories or IM instruments and used as evidence for the identification of unknown ions.

We have previously demonstrated the feasibility of hydrophilic interaction liquid chromatography (HILIC) and IM-MS to resolve four representative Gram-negative and Gram-positive bacteria based on the simultaneous analysis of lipids and metabolites from single-phase extracts.^37^ However, the redundancy of the class-level separation in the HILIC and IM dimensions suggests that one pre-MS separation dimension may be sufficient for the analysis of bacterial lipids and metabolites. Examples of rapid IM-MS measurements without chromatographic separations have been achieved by others using sample introduction strategies like paper spray (PS)^26,27^ and flow injection (FI).^38–41^ These studies have primarily focused on narrow subsets of biomolecules, such as metabolites or lipids, and have not investigated the impact of these sample introduction strategies on the overall performance relative to methods that use chromatographic separations. Here, we perform a side-by-side comparison of HILIC-IM-MS and FI-IM-MS for strain-level differentiation in six types of bacteria based on their multiomic profiles. Our study demonstrates that FI-IM-MS can achieve the same quality of data as HILIC-IM-MS in a fraction of the time. The ability to perform rapid analyses of bacterial lipids and metabolites, enabled by IM-MS and FI, represents a positive step toward the development of multiomic methods that have the speed and throughput required for clinical microbiology and other diagnostic areas.

## Experimental Methods

### Bacteria Species and Culture Conditions

A total of 24 strains (Document S2) were used in this study to represent the six different species of Gram-positive (*n* = 2) and Gram-negative (*n* = 4) bacteria that comprise the ESKAPE pathogens. All work with microorganisms was performed under Biosafety Level 2 (BSL-2) conditions. Bacteria were streaked onto Bovine Heart Infusion agar plates from stocks and incubated overnight at 37 °C. Single colonies were collected from the agar plates and suspended in sterile deionized (DI) water to a turbidity of 2.0–2.05 McFarlands (equivalent to ca. 6.0 × 10^8^ CFUs/mL). Four biological replicates were prepared for each strain. Tryptic Soy Broth was inoculated at a 1:10 dilution (5 mL total volume) and incubated overnight at 37 °C with shaking (200 rpm). The cultures were then centrifuged at 2700 rpm for 10 min at 4 °C, after which the broth was discarded. The pelleted bacteria were washed and resuspended in 2 mL of sterile water and prepared for single-point optical density measurements using an Epoch 2 Absorbance Microplate Spectrophotometer (Document S2).

### Solvent and Authentic Standards

Phosphatidylcholine (PC) and phosphatidylethanolamine (PE) standards were acquired from Avanti Polar Lipids. Acetaminophen, caffeine, carnosine, d-Glucose 6-phosphate, *S*-(5′-adenosyl)-l-methionine (SAM), and poly-dl-alanine were purchased from Sigma-Aldrich. Adenosine 5′-monophosphate (AMP), adenosine 5′-diphosphate (ADP), 2-heptyl-3-hydroxy-4­(1H)-quinolone (PQS), and pyocyanin were purchased from Cayman Chemical. l-Histidine, l-phenylalanine, sucrose, ammonium formate, formic acid, HPLC-grade water (H_2_O), acetonitrile (ACN), methanol (MeOH), and 1-butanol (BuOH) were purchased from Thermo Fisher Scientific.

### Extraction Methods

Prior to extraction, the washed pellets were reconstituted in 0.5 mL of cold HPLC-grade H_2_O, which were then transferred to 1.5 mL microcentrifuge tubes before being centrifuged at 3500 rpm at 4 °C to separate the supernatant. Extractions were performed by adding 1 mL of a chilled solution containing 45% BuOH/35% ACN/20% H_2_O (45% BAW) to the bacterial pellets.^37^ After 5 min of vortexing, the samples were chilled at 4 °C for 10 min and then centrifuged at 4000 rpm at 4 °C for 10 min. Supernatants were collected into 2 mL LC-autosampler vials and dried under vacuum. The dried single-phase extracts were reconstituted in 200 μL of 2:2:1 ACN/MeOH/H_2_O and stored at −80 °C until analysis.

### Sample Preparation

For FI and HILIC analyses, aliquots of the bacterial extracts were transferred to fresh autosampler vials and diluted 5× with an additional 2:2:1 ACN/MeOH/H_2_O. A quality control sample (QC) was prepared by pooling 5 μL from one representative extract per strain. An aliquot of the pooled QC sample was diluted 5× with 2:2:1 ACN/MeOH/H_2_O for analysis. Solutions of pyocyanin were prepared at 30 nM in neat 2:2:1 ACN/MeOH/H_2_O and in a 5× dilution of strain NR-52183. The autosampler chamber was maintained at 6 °C, and an injection volume of 5 μL was used for all samples.

### Ion Mobility–Mass Spectrometry

Hydrophilic interaction liquid chromatography (HILIC) and flow injection (FI) were coupled to the electrospray ionization source of a Waters SYNAPT XS traveling wave ion mobility mass spectrometer (TWIM-MS) for data collection. Sample introduction methods and mass spectrometry settings can be found in Document S1. All collision cross section (CCS) calibration methods and results can be found in the Supplemental Experimental Methods section of Document S1. Details on the components and concentrations of the CCS calibration mixtures can be found in Document 2. Data were analyzed using Water’s Driftscope, Progenesis QI, and EZInfo. For FI-IM-MS data, Driftscope was used to collapse the retention time dimension and export the corresponding drift time dimension or arrival time distribution. An example of the resulting IM-MS data, arrival time distribution, and representative extracted mobilograms are shown in Figure 1. Progenesis QI was then used for both FI-IM-MS and HILIC-IM-MS data sets to import, peak pick, align, normalize, and visualize the data. All data were normalized to the OD readings during the bacterial growth process (Document S2). The data were then filtered by ANOVA *p*-value or by feature intensity before being exported to EZInfo for principal component analysis (PCA). Student’s *t*-test *p*-values were corrected for multiple comparisons using the Benjamini-Hochberg method.^42^ The raw HILIC-IM-MS and FI-IM-MS data files are available in the MassIVE data repository (MSV000097587). Additional information on the identification of lipid and metabolite features using accurate mass and CCS can be found in Document S1.

**Figure 1:**
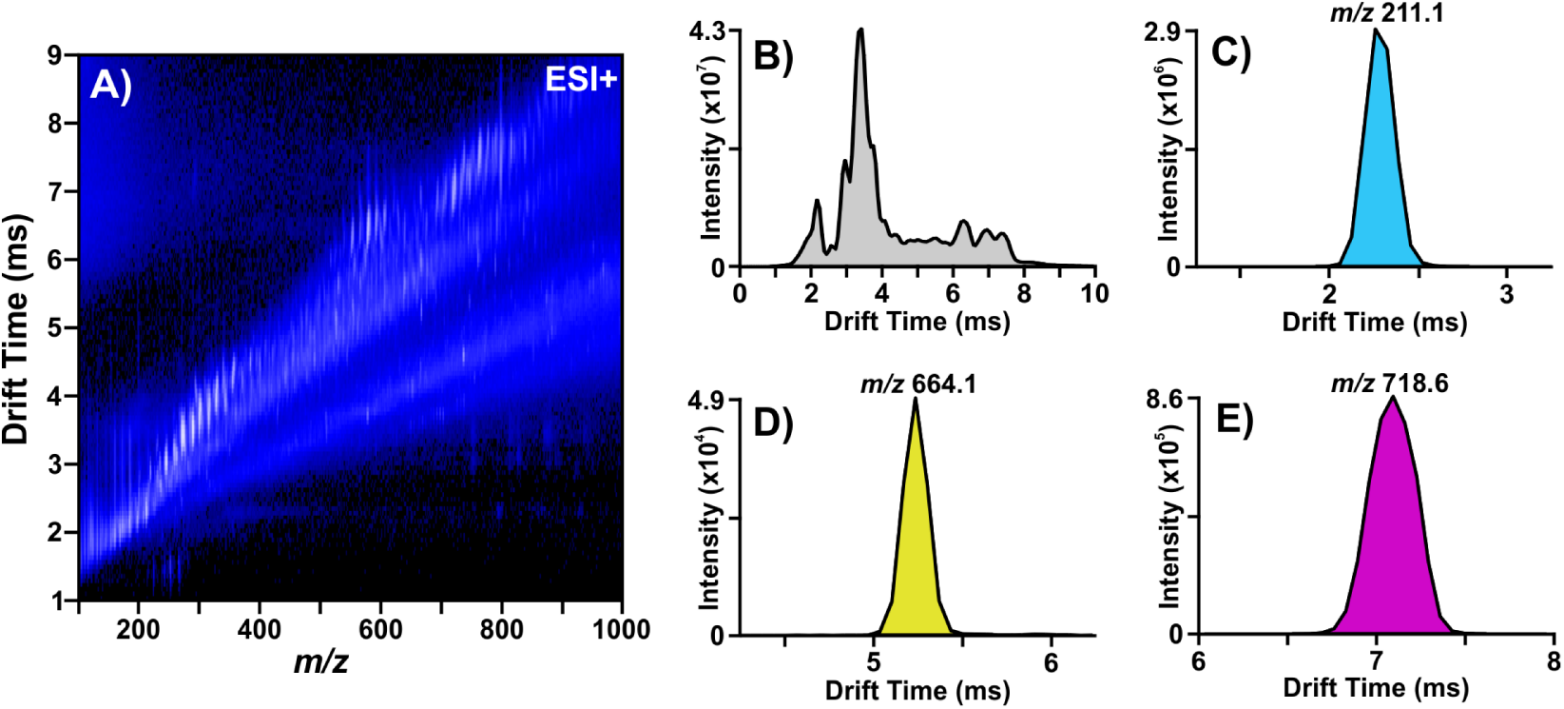
FI-IM-MS (ESI+) data from multiomic mixture containing strain NR-51529. (A) Ion mobility–mass spectrometry conformational space. From the ion mobility dimension, (B) the total 1D ion mobilogram and extracted ion mobilograms for (C) pyocyanin, (D) nicotinamide adenine dinucleotide, and (E) phosphatidylethanolamine (PE) 34:1 are shown.

## Results and Discussion

We previously demonstrated the feasibility of HILIC and IM-MS to distinguish four representative Gram-positive and Gram-negative bacteria to the species level using lipids and metabolites isolated in a single-phase extraction method.^37^ While the use of an LC separation enabled the use of routine lipid/metabolomic software, the information within the HILIC dimension itself was largely redundant to the information that was provided from the IM-MS portion of the data set as they both perform broad class-level separation of biomolecules. In the current study, we investigated the feasibility and performance impacts of eliminating the LC separation from our IM-MS multiomics method for bacteria identifications in an effort to reduce analysis time and increase the suitability for a wider range of biomolecules. Our strategy to achieve these goals, we adopted flow injection (FI) to rapidly introduce bacteria extracts to the electrospray ionization source. To determine if FI-IM-MS could yield comparable results to those obtained using LC, we performed head-to-head analyses of 24 ESKAPE pathogen strains by FI-IM-MS and HILIC-IM-MS. Our ESKAPE pathogen collection contained two Gram-positive organisms: and , and four Gram-negative organisms: , , , and (see Document 2). This collection translates to a total of 96 extracts when individual strains (*n* = 4) from each organism and the biological replicates (*n* = 4) are included. Inclusive of both ionization modes, the 2 min FI-IM-MS method required 10 h to collect data for 226 injections whereas the 17 min HILIC-IM-MS strategy required 63 h to collect data for a similar number of injections.

### Precision of FI-IM-MS versus HILIC-IM-MS

Before evaluating the ability to distinguish bacterial species, we first investigated the precision of the FI-IM-MS method relative to our previously demonstrated HILIC-IM-MS approach. Precision was evaluated using the IM-extracted peak areas of features detected in replicate injections of a pooled quality control (QC) sample that contained a mixture of extracts from each strain evaluated. As seen in Figure 2A,B, FI-IM-MS data collected for 16 QC injections over a 5-h period cluster tightly in the PCA score plots without any need for filtering. The relative standard deviation (%RSD) of FI-IM-MS features with peak intensities greater than 50 was calculated. This threshold retained 80% (1481/1736) and 85% (1394/1739) of the total detected features from positive and negative ionization modes, respectively. As seen in Figure 2C,D, 79% and 90% of the retained FI-IM-MS features had RSDs below 15% in positive ionization mode and negative ionization mode, respectively. These data demonstrate that the FI-IM-MS method and particularly the IM dimension from which peak areas were extracted provide high-precision measurements for complex biological extracts in a short analysis time.

**Figure 2:**
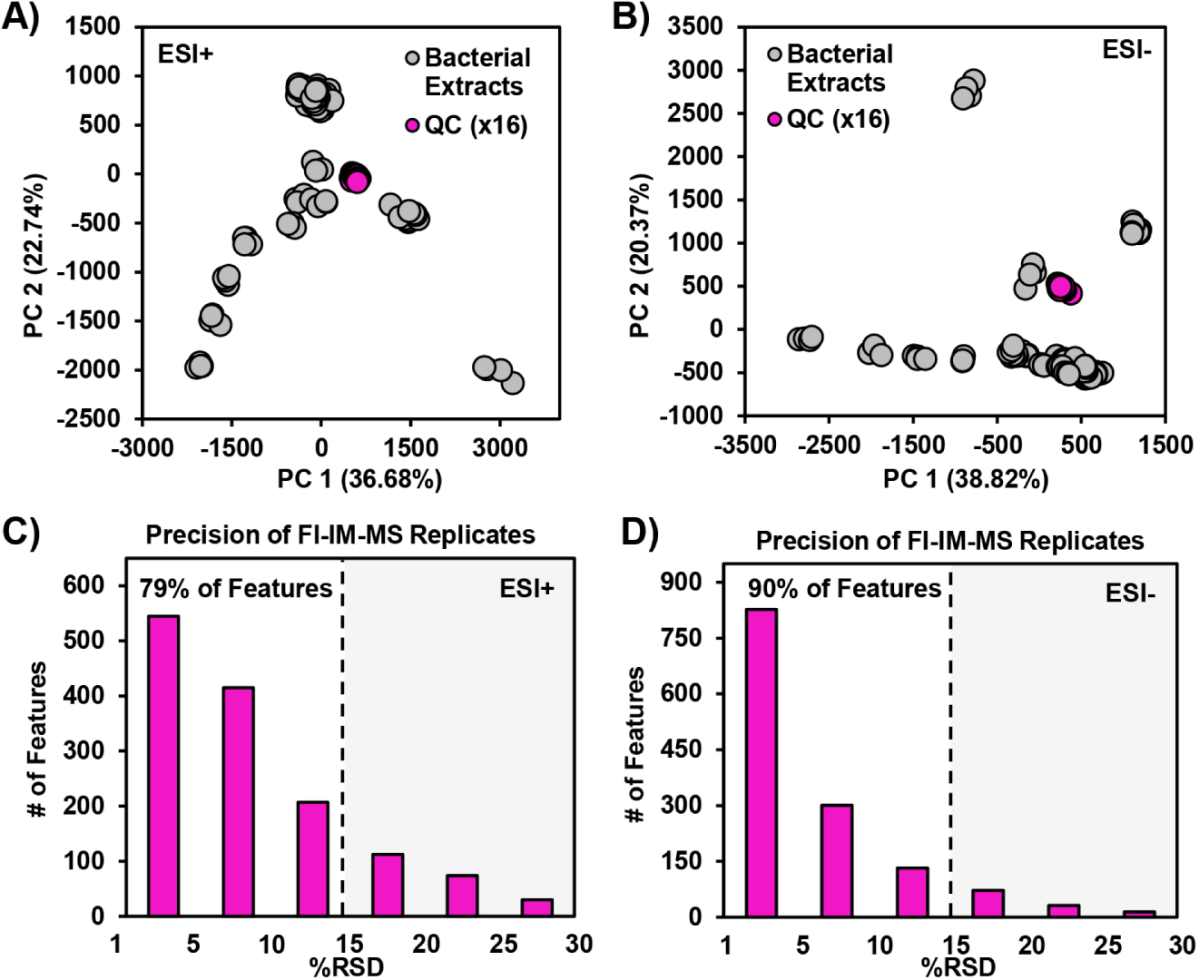
PCA plots of (A) positive and (B) negative mode FI-IM-MS data from 16 injections of a pooled QC and 96 bacteria extracts. RSDs calculated based on (C) 1481 features in ESI+ and (D) 1394 features in ESI– that were detected in all QC replicates with an intensity ≥50.

For comparison, we performed the same evaluation on LC-extracted peak areas of features detected in 10 replicate injections of QC collected throughout the HILIC-IM-MS experiment. The combination of a 17-min HILIC separation with IM-MS provides a higher peak capacity than the 2 min FI-IM-MS method, and therefore more features were detected in the HILIC-IM-MS data set.^43,44^ The same intensity threshold of 50 retained 38% (4165/10953) and 20% (1434/7080) of the respective positive and negative mode HILIC-IM-MS features. Although the percentage retained is smaller, this filtered HILIC-IM-MS data set still represents a similar magnitude of features (1× for negative mode and 3× more for positive mode) compared to the FI-IM-MS data set. As shown in Document S1, Figure S3, just 60% of the positive mode HILIC-IM-MS features and 54% of the negative mode HILIC-IM-MS features had RSDs below 15%.

We then evaluated the precision of the two methods based on a set of features that were detected in both analyses. Each data set was filtered to retain the top 1400 most abundant features, after which the data sets were aligned by *m*/*z* and CCS to identify features that were detected in both data sets. For positive mode, we found 472 features that were shared between the FI-IM-MS and HILIC-IM-MS data sets (see Figure S4 in Document S1 and Document S2 for list format). For negative mode, there were 490 overlapping features between the two data sets. The PCA score plots based on just several hundred features (Figures S5 and S6 in Document S1) show clustering of the QC injections that is similar to the PCA score plots based on several thousand features (Figure 2 and Figure S3 in Document S1). However, the differences in the precision of FI-IM-MS versus HILIC-IM-MS peak areas persisted. For FI-IM-MS, 88% (ESI+) to 91% (ESI−) of the features detected in both analyses had RSDs less than 15% (Figure S5 in Document S1). On the other hand, 75% (ESI+) and 63% (ESI−) of the HILIC-IM-MS features detected in both analyses had RSDs less than 15% (Figure S6 in Document S1). Based on the same set of features, the FI-IM-MS data set demonstrated substantially higher precision than the HILIC-IM-MS data set. This can be attributed to the excellent stability and precision of gas-phase separations, as well as the shorter run times required for the FI-IM-MS method compared to HILIC-IM-MS (i.e., 10 h vs 63 h).

Although the overlapping features were selected from the QC samples, these 472 and 490 features are sufficient to distinguish the bacterial species and strains represented in the full data set (Figure S7 in Document S1). Over 70% of the variance in the respective data sets is explained within principal components 1 through 3, although slightly higher amounts of variance are explained in the FI-IM-MS data set (75.4% vs 74.8% for positive mode FI and HILIC, respectively; 74.9% vs 71.8% for negative mode FI and HILIC, respectively). These results concur with the evidence of higher technical variability in the negative mode HILIC-IM-MS analysis, which reduces the amount of biological variability that can be explained in the PCA. Overall, the PCAs based on the overlapping features from the FI-IM-MS and HILIC-IM-MS methods demonstrate that FI-IM-MS can achieve similar, if not better, results for the differentiation of bacterial species and strains in a fraction of the time.

### Correlation between FI-IM-MS and HILIC-IM-MS

We next evaluated the correlation between the FI-IM-MS and HILIC-IM-MS data sets to demonstrate that peak areas extracted from the IM dimension of the FI-IM-MS data set show strong agreement with the LC-extracted peak areas from the HILIC-IM-MS data set. The correlation between the two aligned data sets was determined from the natural log-scaled feature abundances (Figure 3). Moderate positive correlation was observed for all overlapping features between FI and HILIC, with *R* ^2^ values of 0.44 and 0.51 in positive and negative ionization modes, respectively (Figure 3A,D). However, the correlation between the two data sets is stronger when considering features for which identifications have been obtained (see Document S2 for lists of aligned features and identifications). The *R* ^2^ values for metabolites detected in positive and negative modes (*n* = 19 and *n* = 16, respectively) demonstrate stronger positive correlation (Figure 3B,E) between the two methods (ESI+, *R* ^2^ = 0.84; ESI–, *R* ^2^ = 0.84, respectively). This agreement is even stronger among the lipid species that are present within both data sets (Figure 3C,F), with *R* ^2^ values of 0.93 and 0.89 for lipids in positive (*n* = 31) and negative (*n* = 34) modes, respectively. Evidence of strong positive correlations between the identified lipids and metabolites that are shared between the FI-IM-MS and HILIC-IM-MS data sets indicates that FI-IM-MS can detect the same features as the HILIC-IM-MS method and with similar relative abundances.

**Figure 3:**
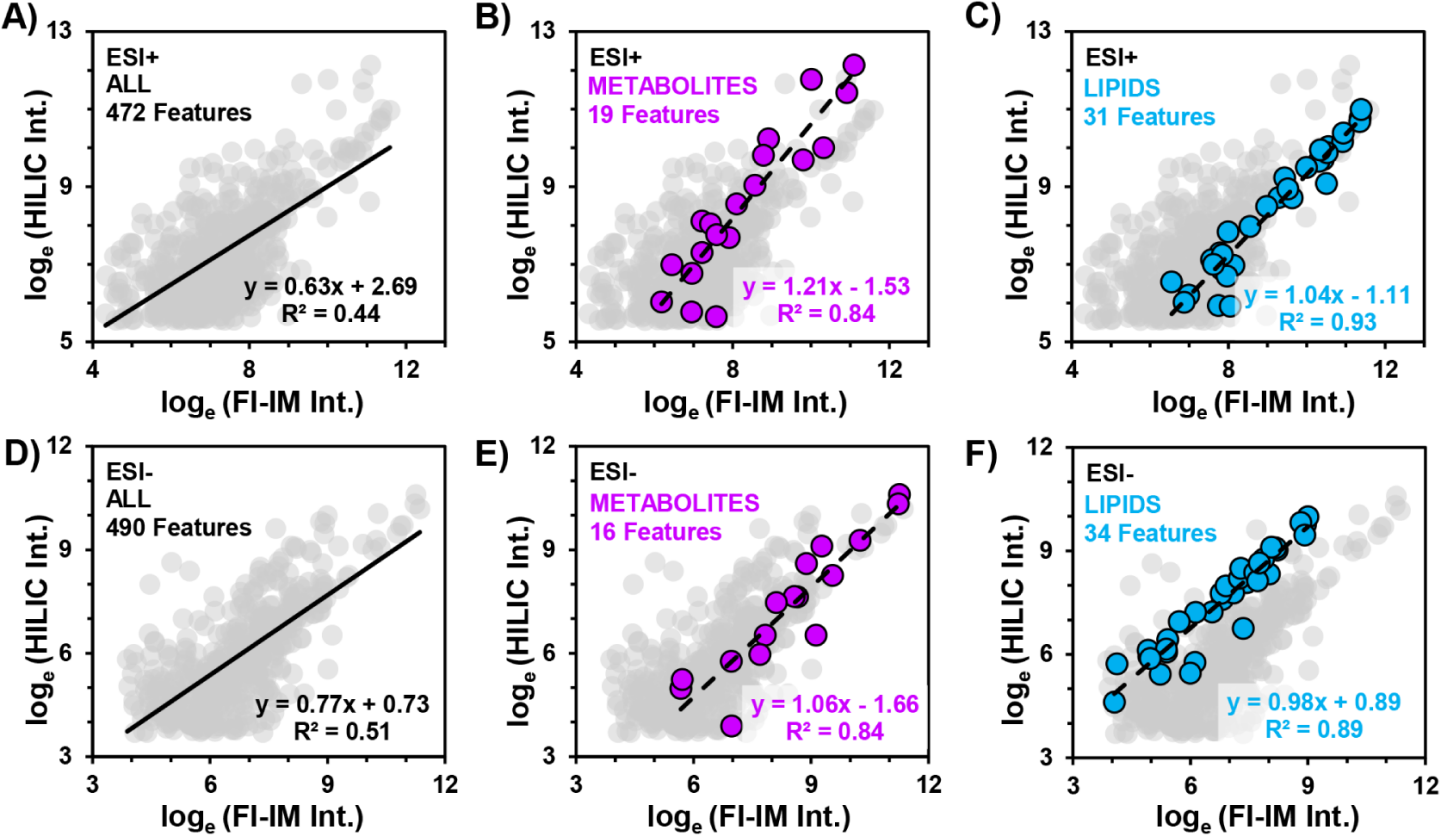
Correlation plots of feature intensities determined by HILIC-IM-MS versus intensities determined by FI-IM-MS. Data were collected in both positive (A–C) and negative (D–F) ionization modes and scaled by natural log (log_e_).

Ionization suppression is one of the concerns that is cited for direct sample introduction methods like FI, but it is difficult to quantify for all molecules that are present in a complex sample.^45^ The influence of a complex biological matrix on ionization following FI and HILIC is demonstrated in Figure 4 using the small metabolite pyocyanin, which contributes to the differentiation of from other Gram-negatives. Pyocyanin is predicted to have high ionization efficiency due to its phenazine core and therefore can provide a good estimation of general ionization suppression influences between the two methods. Pyocyanin solutions were prepared at 30 nM in neat solvent or spiked into an extract of , which does not produce this pigment. The overall intensity of the pyocyanin signal is higher in the HILIC data than in the FI data in both the neat and spiked conditions. However, both sample introduction strategies clearly suffer from significant ionization suppression. The HILIC-IM-MS analysis revealed a 76% decrease in the signal intensity of pyocyanin when it was analyzed in the presence of the extract. The pyocyanin intensity was reduced even further in the FI-IM-MS analysis, with an 87% reduction in the presence of the extract compared with the neat solution. These data demonstrate that ionization suppression effects are of a similar magnitude for FI-IM-MS and HILIC-IM-MS for an easily ionized small molecule. However, analytes that ionize less efficiently or occur at lower concentrations may be difficult to detect with HILIC-IM-MS or below the detection limits of FI-IM-MS.

**Figure 4:**
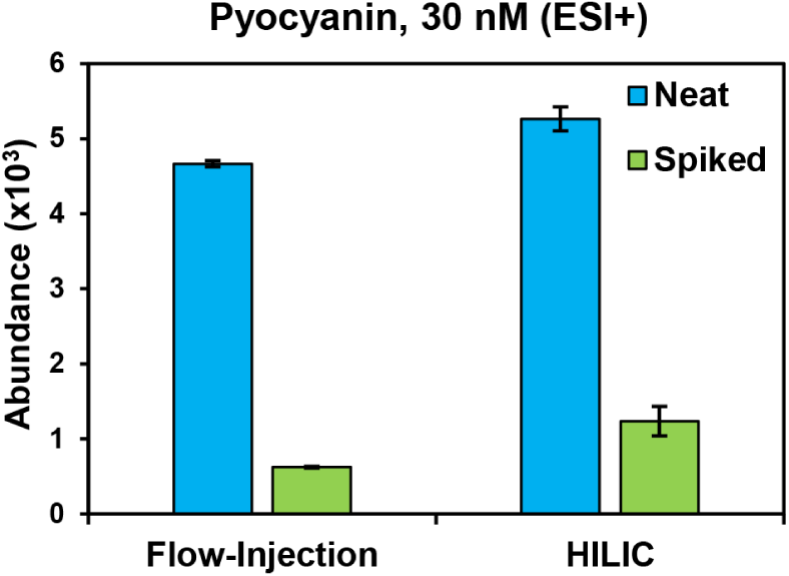
Abundance of pyocyanin from FI-IM-MS and HILIC-IM-MS of 30 nM solutions with and without bacterial extract. The neat solution was prepared in 2:2:1 ACN/MeOH/H_2_O and analyzed in technical triplicate. The spiked solution was prepared by adding pyocyanin into diluted extracts of strain NR-52183 biological replicates.

### Applying FI-IM-MS to Distinguish Bacterial Species and Strains

While ionization suppression may lead to the absence of some low-abundance HILIC-IM-MS features in the FI-IM-MS data set, the more pertinent performance evaluation is whether the FI-IM-MS method can achieve species- and strain-level separations based on the features it is able to detect. Figure 5 contains the results from the independent analysis of FI-IM-MS measurements for the 24 bacterial strains and their biological replicates. The PCA score plots shown in Figure 5A,B are based on the full FI-IM-MS data sets containing 1736 features in positive mode and 1739 features in negative mode, and each represents approximately 70% of the variability in the data set in the first three components. Qualitatively, the PCA plots depict tight clustering of biological replicates as well as separation of the organisms by Gram-stain classification (Gram–, G–; Gram+, G+), species, and strain. Strain-level separation within each species is maximized by the FI-IM-MS data set when processed and filtered independently from the HILIC-IM-MS data set (see Figure S8 in Document S1). The volcano plot in Figure 5C demonstrates that both metabolites and lipids contribute to the separation of Gram+ and Gram– bacterial species, which is consistent with both these and previous results from HILIC-IM-MS. Evidence to support the lipid and metabolite identifications can be found in Document S2, and their trends in IM-MS space can be found in Figure S9 (Document S1). Among the identified features, many prominent lipids and metabolites that contribute to the separation by Gram-stain classification are in the top 10% of statistically significant features for both the FI-IM-MS and HILIC-IM-MS data sets (Document S1, Figure S10). For example, both sets had the same 11 PEs, 3 PCs, 14 DGs, 2 DGDGs, 1 LysylPG, Pyocyanin, 5 quorum-sensing molecules, and 14 metabolites that showed up as statistically significant. Specifically, both had DG 32:1, DG 36:2, PE 32:1, PE 36:2, choline, and nicotinamide adenine dinucleotide in the top 10% of most statistically significant features. The only noticeable difference was the absence of several LysylPGs and lysolipids in the FI-IM-MS data set. Based on the low abundance at which they were detected in the HILIC data set, their absence in the FI-IM-MS data set is likely due to the ionization suppression effects discussed above.

**Figure 5:**
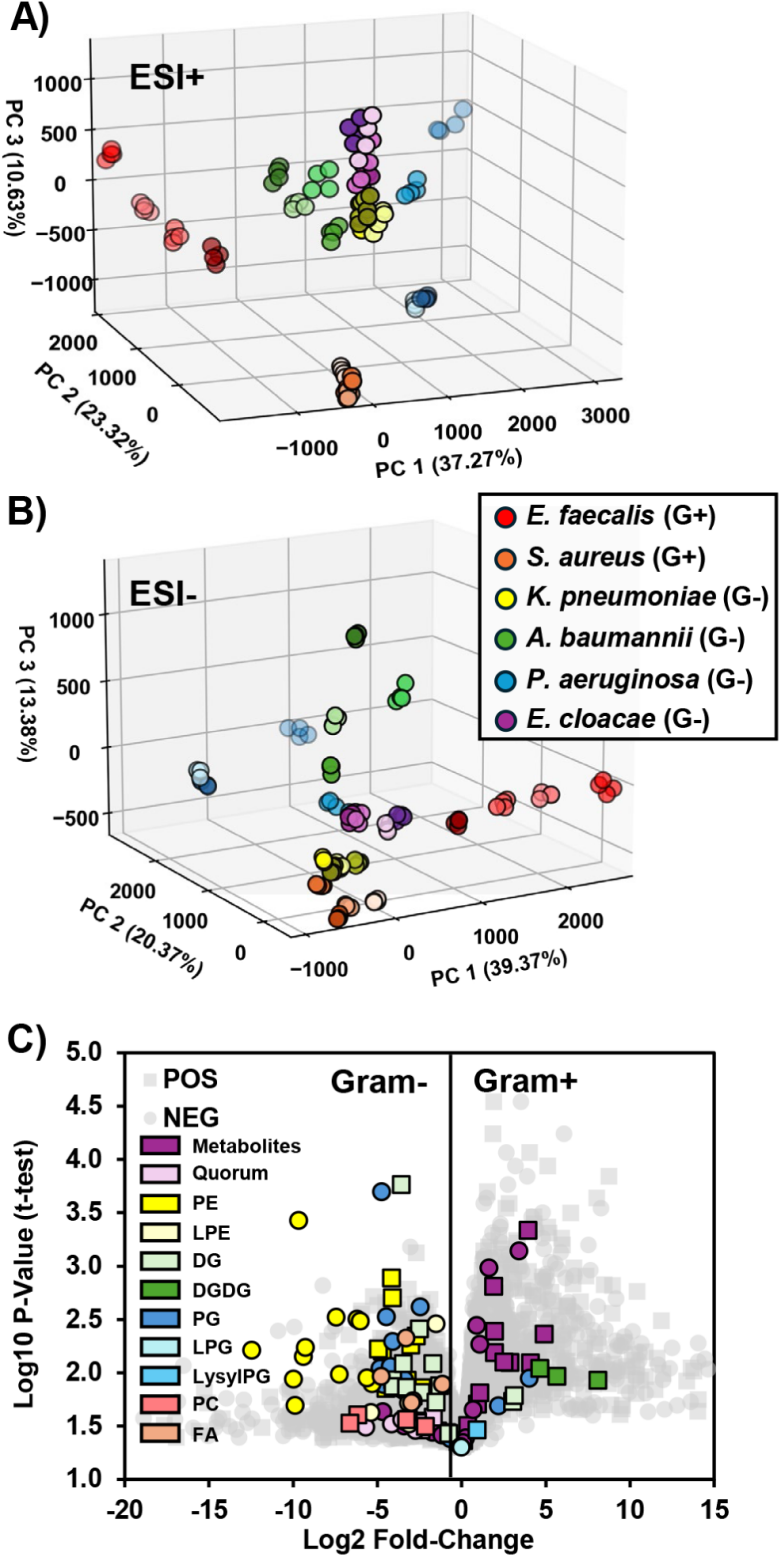
PCA score plots from FI-IM-MS measurements of 24 bacteria strains collected in (A) ESI+ and (B) ESI– modes. Score plots are based on approximately 1740 features in each mode. Colors correspond to the organism, and shading corresponds to strains. (C) Volcano plot of features detected by FI-IM-MS in positive (square) and negative (circle) mode analyses of Gram-positive and Gram-negative bacterial extracts. Identified compounds are colored based on their biomolecular classes. Student’s *t*-test (two-way, unpaired) *p*-values were corrected for multiple comparisons with the Benjamini-Hochberg method.

**Figure 6:**
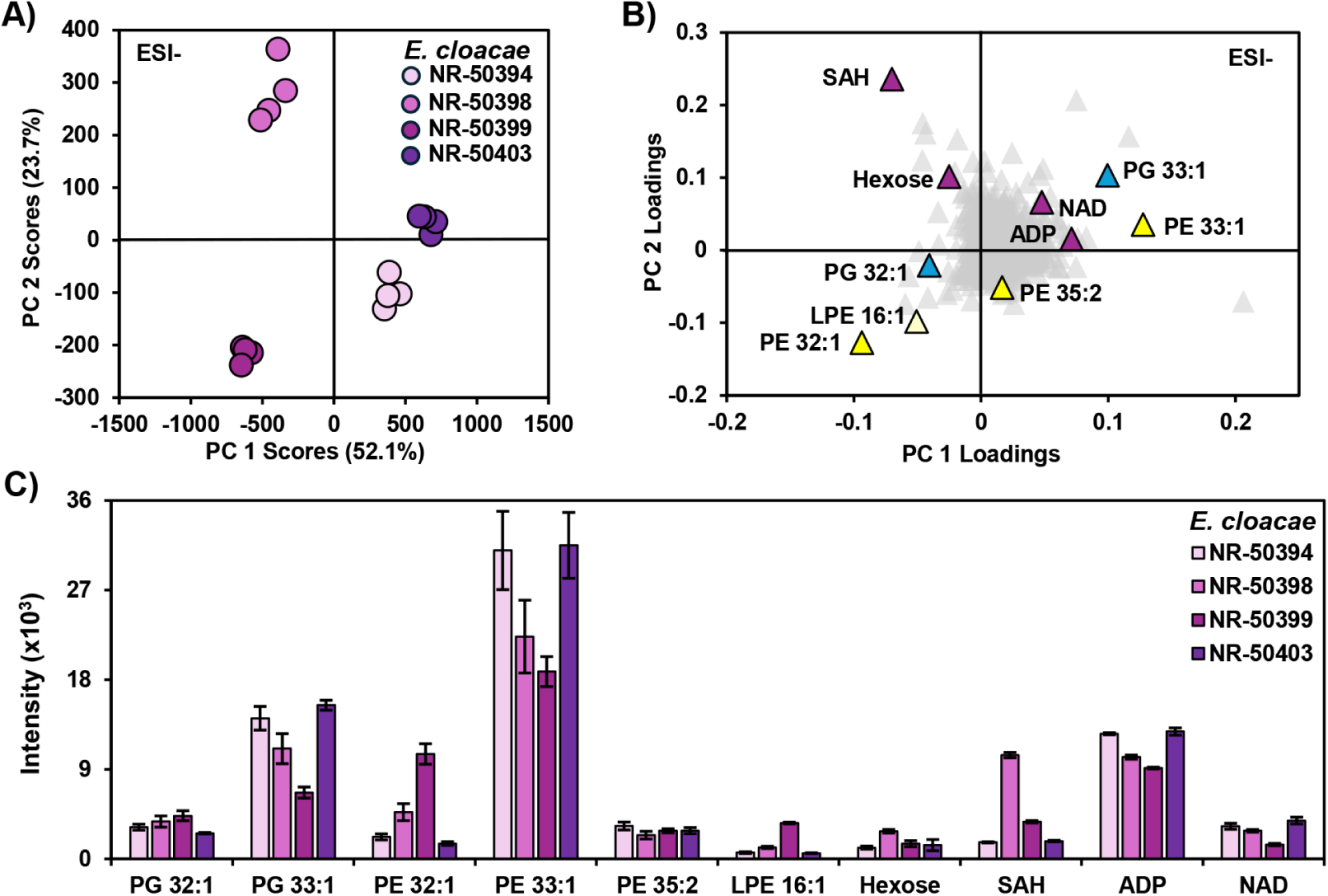
(A) PCA scores plot and (B) PCA loadings plot of four strains run in quadruplicate analyzed by FI-IM-MS in negative ionization mode. (C) Bar chart of average abundances of important identified compounds highlighted in the loadings plot. Bars are colored based on their corresponding strain as seen in (A).

To illustrate the strain-level distinctions that can be made from the FI-IM-MS data, Figure 6 highlights the results for the four strains of . The four strains can be separated along two principal components, with the separation of NR-50394 and NR-50403 from NR-50398 and 50399 along PC1 (Figure 6A). The close spacing of NR-50394 and NR-50403 along PC1 and PC2 suggest that they have very similar molecular profiles. On the other hand, NR-50398 and NR-50399 are resolved from each other along PC2, which suggests that they are more biochemically similar to one another than NR-50394 and NR-50403, while having molecular differences that differentiate them. The exact molecular species responsible for the separations observed in the PCA can be identified from the loading plot (Figure 6B), which includes both lipid and metabolite species. For example, the metabolite *S*-adenosyl-l-homocysteine (SAH) is in the upper left corner of the loading plot, corresponding to the position of NR-50398 on the scores plot. Looking at the intensity of the identified features across the four strains, SAH is substantially higher in NR-50398 than in any of the other three strains, with NR-50399 the second highest (Figure 6C). These strains did not undergo whole genome sequencing prior to their submission to BEI Resources; however, antimicrobial resistance (AR) results are reported for all strains acquired from BEI Resources. In investigating the AR profiles of the four complex strains, we determined that the primary differences were in the fluoroquinolones, trimethoprim/sulfamethoxazole, and ampicillin (see Document S2). Strains NR-50398 and NR-50399 are both resistant to ciprofloxacin, levofloxacin, and trimethoprim/sulfamethoxazole, whereas NR-50394 and NR-50403 are susceptible to these antimicrobials. Although it is not possible to determine the exact determinants of resistance without genetic sequencing, there are reports in the literature to indicate a relationship between ciprofloxacin resistance and methionine metabolism, including the methionine metabolite SAH.^46^ Many other lipid and metabolite features in the data set, and the strain-level data sets for the five other bacterial species are still to be investigated (Document S1, Figures S11 and S12). However, these results suggest that it is feasible to resolve strain-level differences, and perhaps AR profile differences, using the FI-IM-MS strategy for rapid multiomics of bacteria.

## Conclusions

We have demonstrated that the IM-MS strategy for multiomics can achieve species- and strain-level separations for representative strains of the ESKAPE pathogens with an analysis time of 2 min per injection. The FI-IM-MS approach has proven to provide results comparable to those of a previous method for simultaneous lipid and metabolite measurements in bacteria, which was based on a 17-min HILIC-IM-MS method. Although the FI-IM-MS method is more susceptible to ionization suppression, we have demonstrated that the impact of ionization suppression on an easily ionized metabolite is similar between FI and HILIC. The large number of overlapping features and strong positive correlations for the raw abundances of identified lipids and metabolites between the two data sets further demonstrates that the FI-IM-MS approach can achieve similar performance to the HILIC-IM-MS method, but in a fraction of the time. Collectively, these results represent a promising first step in the development of rapid IM-MS-based multiomics as a platform for bacterial identification. Future work will require the curation of databases containing multiomic bacterial signatures and the establishment of scoring algorithms that can be used to identify unknown bacterial isolates. We expect that IM-MS-derived CCS values will be critical in ensuring that bacterial identifications are based on accurately annotated features.

## Supplementary Material





## Notes

Document 1: Additional experimental methods for CCS calibration, HILIC-IM-MS, and FI-IM-MS; additional results for FI-IM-MS and HILIC-IM-MS analysis of bacterial strains (PDF)
Document 2: Additional details on sources of bacterial strains used in this study; information for lipids and metabolites identified in this study (XLSX)
