# Supplementary material for: Rapid Multi-Omics for Bacterial Identification Using Flow Injection–Ion Mobility–Mass Spectrometry
Source: Anal Chem. 2025 Jun 24;97(26):13809–16. doi: 10.1021/acs.analchem.5c00417 (PMC12242902; doi:10.1021/acs.analchem.5c00417)
Supplement: Supplementary file 1 [file ac5c00417_si_001.pdf]

Supporting Information

Rapid Multi-Omics for Bacteria Identifications using Flow Injection-Ion Mobility-Mass Spectrometry

Hannah M. Hynds, Jana M. Carpenter, and Kelly M. Hines*

Department of Chemistry, University of Georgia, Athens, Georgia 30602, United States

*Correspondence should be addressed to: Dr. Kelly Hines, kelly.hines@uga.edu

TABLE OF CONTENTS **Page**

Supplemental Experimental Methods S-3

Figure S1. CCS vs. *m/z* Regression Lines S-5

Figure S2. CCS Calibration Curves S-6

Supplemental Results & Figures S-7

Figure S3. HILIC-IM-MS PCA and RSD Plots for Pooled QC S-7

Figure S4. FI-IM-MS and HILIC-IM-MS Overlap Venn Diagrams S-8

Figure S5. FI-IM-MS PCA and RSD Plots for Overlapping Features in Pooled QC S-9

Figure S6. HILIC-IM-MS PCA and RSD Plots for Overlapping Features in Pooled QC S-10

Figure S7. FI and HILIC PCA Plots of Bacteria Strains based on Overlapping Features S-11

Figure S8. FI-IM-MS PCA Plots of Bacteria Strains based on ANOVA P-value S-12

Figure S9. CCS vs. *m/z* Plots for Annotated Bacteria Lipids and Metabolites S-13

Figure S10. Volcano Plot for Gram-Negative vs. Gram-Positive Comparison of HILIC Data S-14

Figure S11. PCA Plots of Individual Strains by Species, Positive Mode FI-IM-MS S-15

Figure S12. PCA Plots of Individual Strains by Species, Negative Mode FI-IM-MS S-16

Supporting Information References S-17

SUPPLEMENTAL EXPERIMENTAL METHODS

CCS Calibration Methods. Calibration mixtures were prepared using combinations of lipid, small molecule, and peptide standards (see SI Document 2 for list and concentrations). The lipid mixture was prepared in MeOH, 0.1% formic acid. A 5X dilution of the lipid mixture was used for positive ionization mode and a 2X dilution was used for negative ionization mode. The peptide mixture and the small molecule mixture were both prepared in 1:1 MeOH/H_2O, 0.1% formic acid. Data were collected for the lipid, small molecule, and peptide CCS mixtures via 2-minute flow injection runs before collecting extract data. The data was then analyzed using the Multiomic CCS Calibrator (MOCCal)[1] for power-law CCS calibration of the unknown features from the bacterial extracts. The positive mode data were analyzed by MOCCal as found in its originating paper.[1] A version of MOCCal was created for negative mode data, using the CCS Compendium regression lines found in **Figure S1.** The calibration curve parameters and plots of $^{TW}CCS_{N2}$ vs *m/z* of the calibrants for the FI and HILIC experiments can be seen in **Figure S2.** for both positive and negative mode.

Hydrophilic Interaction Liquid Chromatography (HILIC). A single chromatographic method based on hydrophilic interaction liquid chromatography (HILIC) was optimized for the analysis of lipids and metabolites from a single injection. Chromatographic separation was performed on an ACQUITY UPLC BEH Amide column (100 mm x 2.1, 1.7 µm) fitted with a matching precolumn (5 mm x 2.1 mm, 1.7 µm) using a Waters ACQUITY I-Class Plus FTN UPLC system. The column was maintained at 45 °C, with a flow rate of 0.4 mL/min. Solvent A was composed of H_2O with 10 mM ammonium formate and 0.125% formic acid. Solvent B consisted of ACN/H_2O (95/5 *v/v*) with 10 mM ammonium formate and 0.125% formic acid. The gradient, based on Ding et al.,[2] was as follows: 0-2 min at 100% B, 2-7.7 min from 100% to 70% B, 7.7-9.5 min from 70% to 40% B, 9.5-10.25 min from 40% to 30% B, 10.25-12.75 min from 30% to 100% B, and 12.75-17 min to re-equilibrate to 100% B.

Flow Injection. Flow injection was performed on a Waters Acquity FTN I-Class Plus ultraperformance liquid chromatography system using a stainless-steel union and PEEK tubing (1/16 in. o.d. × 0.004 in. i.d. × 2ft, black) and (1/16 in. o.d. × 0.005 in. i.d. × 21.5in, red) in place of a column. A flow rate of 0.2 mL/min was used for 2 min with a constant mobile phase composition of 25% Solvent A (H_2O with 10 mM ammonium formate and 0.125% formic acid), 75% Solvent B (ACN/H_2O (95/5 *v/v*) with 10 mM ammonium formate and 0.125% formic acid).

Mass Spectrometry Data Collection. Data were collected on a Waters SYNAPT XS traveling wave ion mobility mass spectrometer (TWIM-MS) in both positive and negative electrospray ionization mode with the following source conditions: capillary voltage, 3.0 kV (pos)/2.0 kV (neg); sampling cone voltage, 25 V; source offset, 4 V; source temperature, 150 °C; desolvation temperature, 400 °C; desolvation gas flow rate, 900 L/h; cone gas flow rate, 50 L/h. TWIM separations were performed in nitrogen with a gas flow of 90 mL/min, a wave velocity ramp of 600 to 200 m/s, and a wave height of 35 V. Mass calibration was performed with sodium formate over the range of 50-1200 *m*/*z*. The time-of-flight mass analyzer was operated in V mode (resolution mode) with a resolution of ~30,000. Data were collected with a 1 s scan time over the range 50−1200 *m*/*z*. Leucine enkephalin was used for continuous lock-mass correction during acquisition. For FI-IM-MS, data were only acquired for the first minute of the two-minute gradient. For HILIC-IM-MS, MS/MS spectra were acquired using data-independent acquisition (MSe) with a ramped collision energy (from 15 to 45 eV) in the transfer region of the instrument.

Metabolite and Lipid Identifications. Lipid and metabolite identifications were made using *m/z* and MOCCal calibrated $^{TW}CCS_{N2}$ values. Identifications were made against the LipidPioneer,[3] CCS Compendium,[4] CCSBase,[5] and Pseudomonas aeruginosa Metabolome Database (PAMDB) compound libraries. All compound identifications had a mass error tolerance below 10 ppm and CCS errors below 5%.

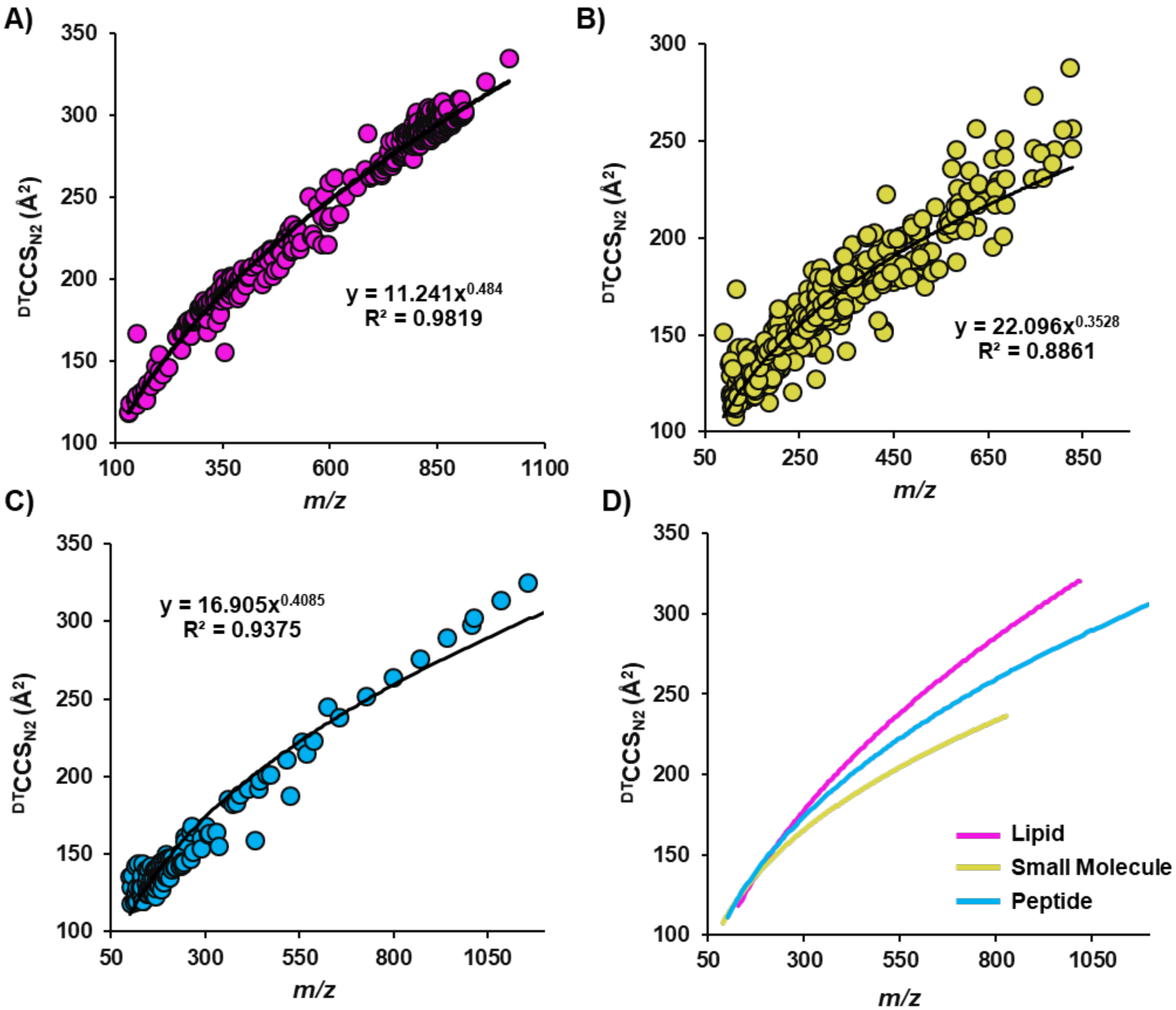

Figure S1. CCS regression lines from the CCS Compendium[4] for negative mode MOCCal[1] calibrations. Regression lines for **(A)** lipids, **(B)** small molecules, and **(C)** peptides. **(D)** Plot of combined regression lines.

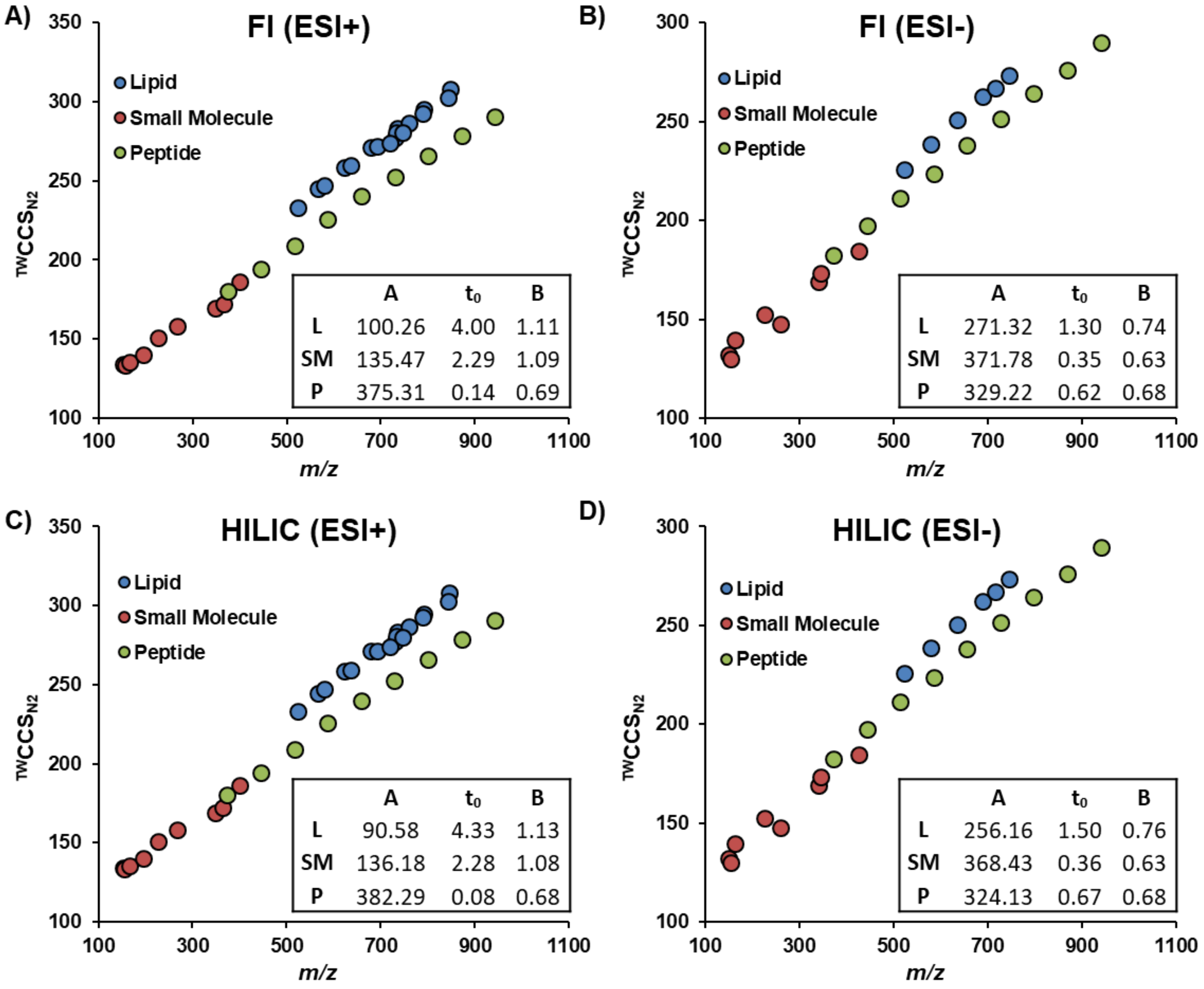

Figure S2. CCS calibration curve parameters and plots of $^{TW}CCS_{N2}$ vs *m/z* values of the lipid (L), small molecule (SM), and peptide (P) calibrants. Calibrations were run before flow-injection experiments in positive **(A)** and **(B)** negative mode and hydrophilic interaction liquid chromatography (HILIC) experiments in positive **(C)** and **(D)** negative mode.

SUPPLEMENTAL RESULTS & FIGURES

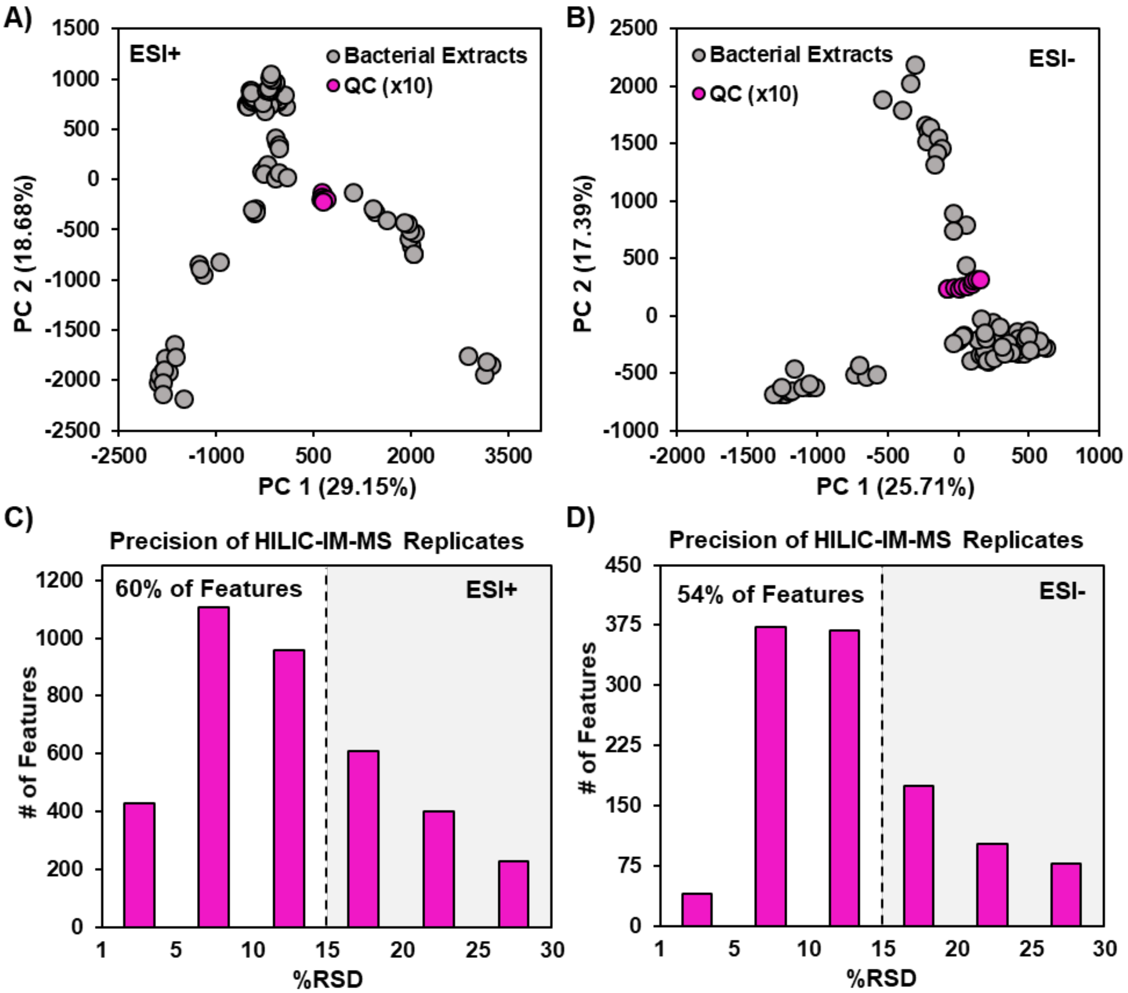

Figure S3. PCA plots of A) positive and B) negative mode HILIC-IM-MS data from 10 injections of a pooled QC and 96 bacteria extracts, after filtering by ANOVA $P \leq 0.5 \times 10^{-5}$. RSDs calculated based on C) 4165 features in ESI+ and D) 1434 features in ESI- that were detected in all QC replicates with an intensity ≥ 50.

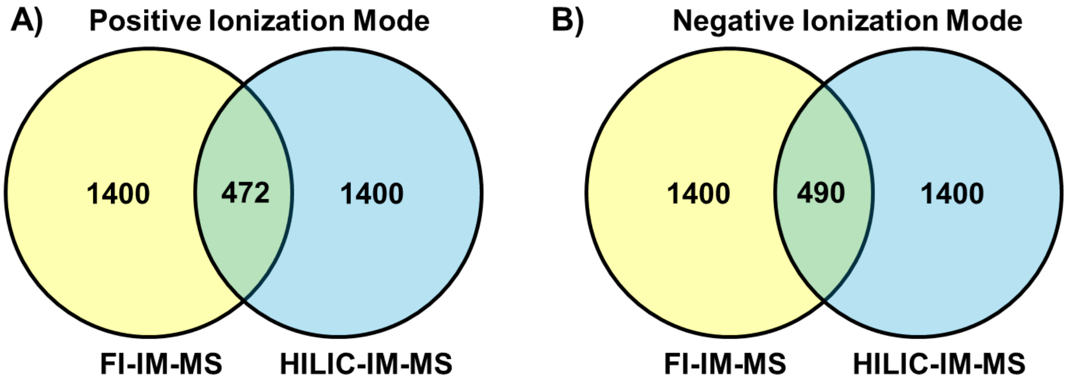

Figure S4. Overview of the overlapping features between the A) positive mode and B) negative mode FI-IM-MS and HILIC-IM-MS datasets. The top 1400 features from the QC samples of both datesets were aligned by *m/z* and CCS to identify the overlapping features.

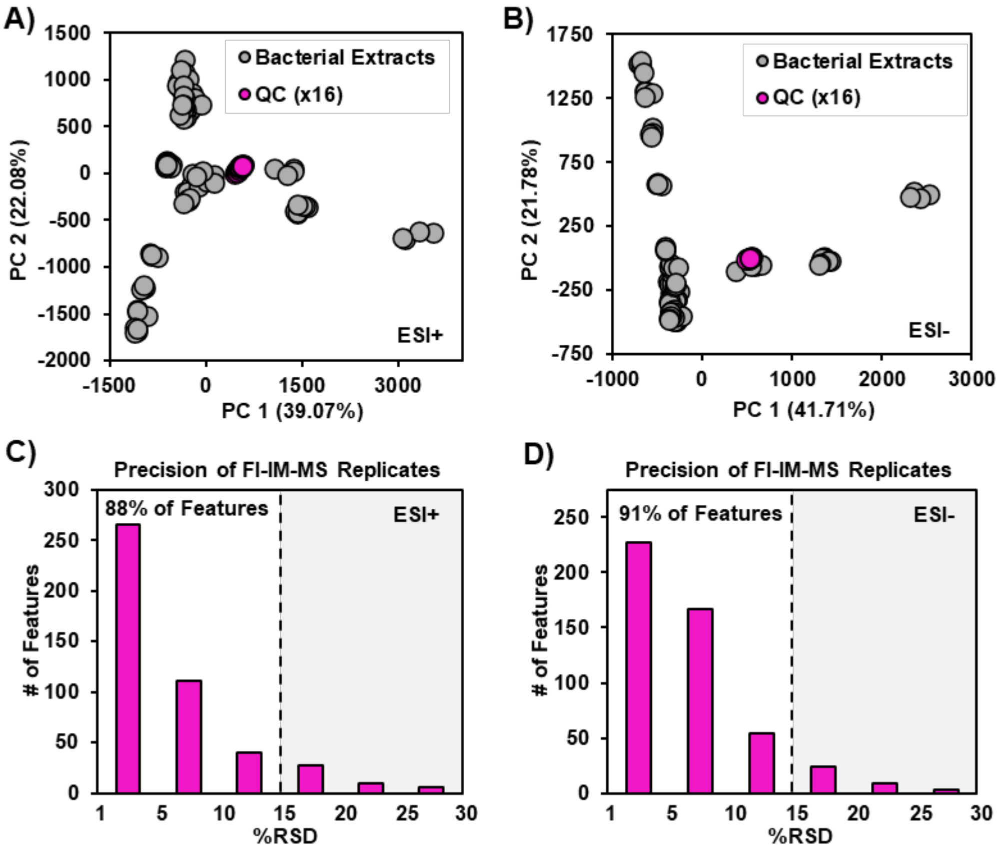

Figure S5. PCA plots of A) positive and B) negative mode FI-IM-MS data from 16 injections of a pooled QC and 96 bacteria extracts. RSDs calculated based on C) 472 features in ESI+ and D) 490 features in ESI- that were detected in the top 1400 features from the QC samples of both datasets.

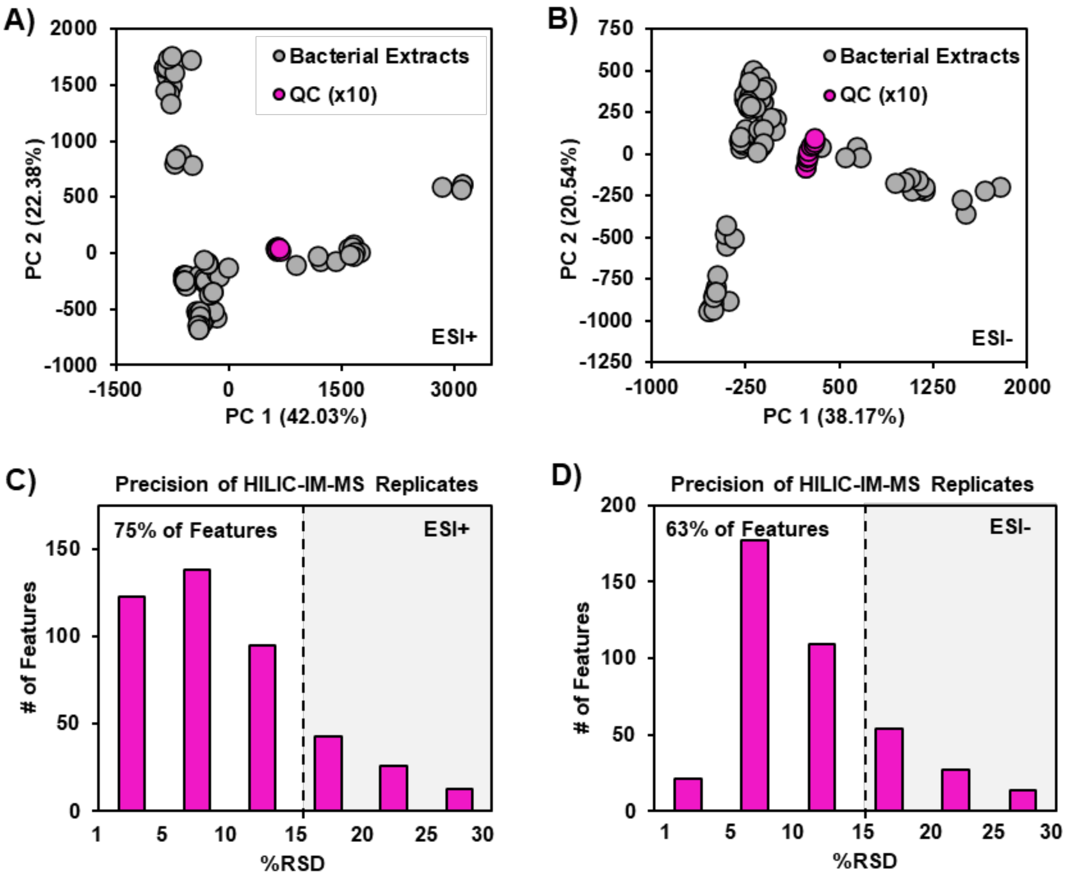

Figure S6. PCA plots of A) positive and B) negative mode HILIC-IM-MS data from 10 injections of a pooled QC and 96 bacteria extracts. RSDs calculated based on C) 472 features in ESI+ and D) 490 features in ESI- that were detected in the top 1400 features from the QCs samples of both datasets.

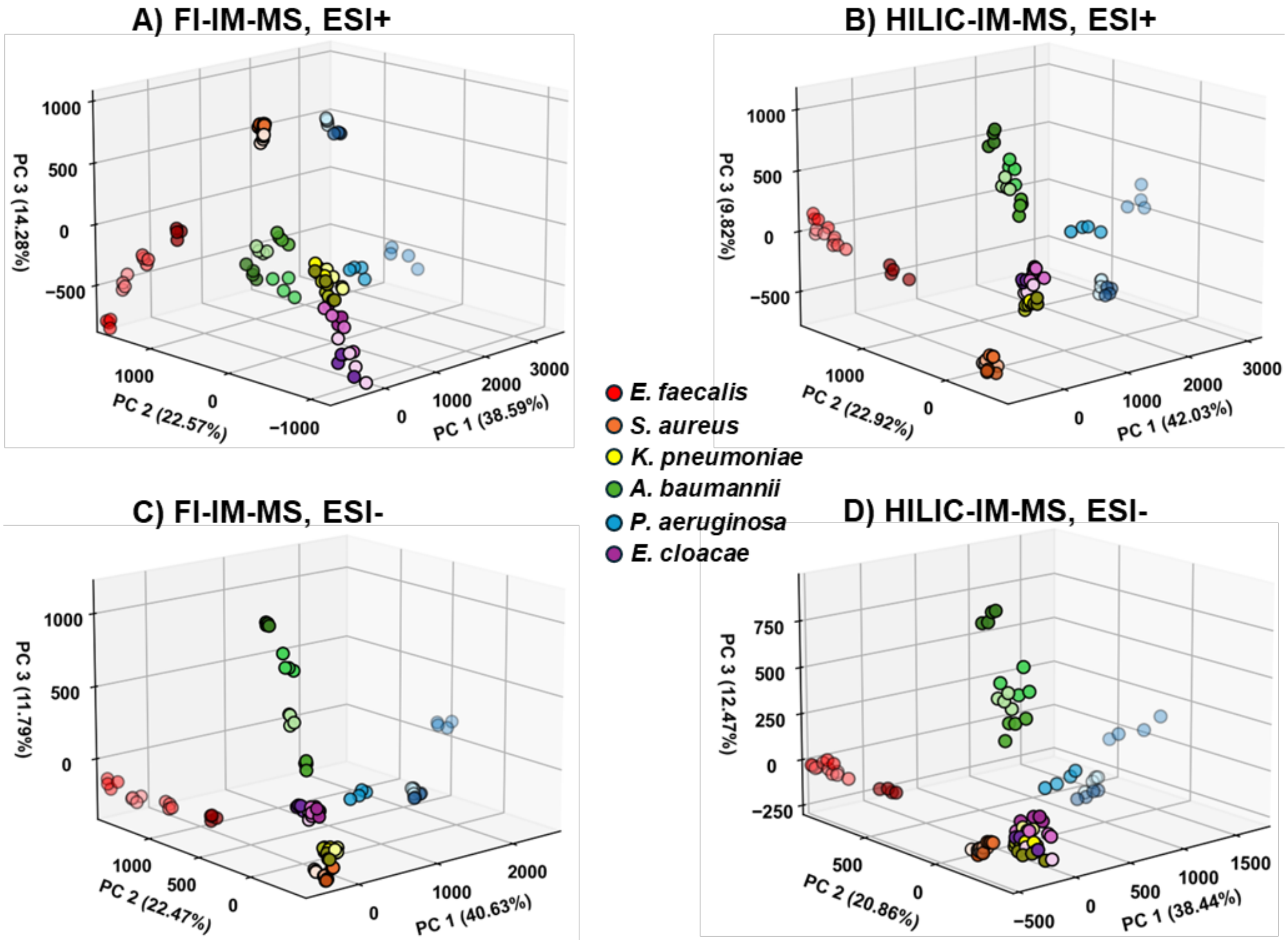

Figure S7. PCA plots of bacterial extracts of 24 strains run in quadruplicate analyzed by FI-IM-MS (**A & C**) and HILIC-IM-MS (**B & D**). PCA was filtered by 472 and 490 overlapping features found in the top 1400 features of the FI and HILIC datasets in positive (**A & B**) and negative (**C & D**) ionization modes, respectively. PCA plot samples are colored with base color of species (6 species) and shade color of strains (4 strains per species, 24 strains).

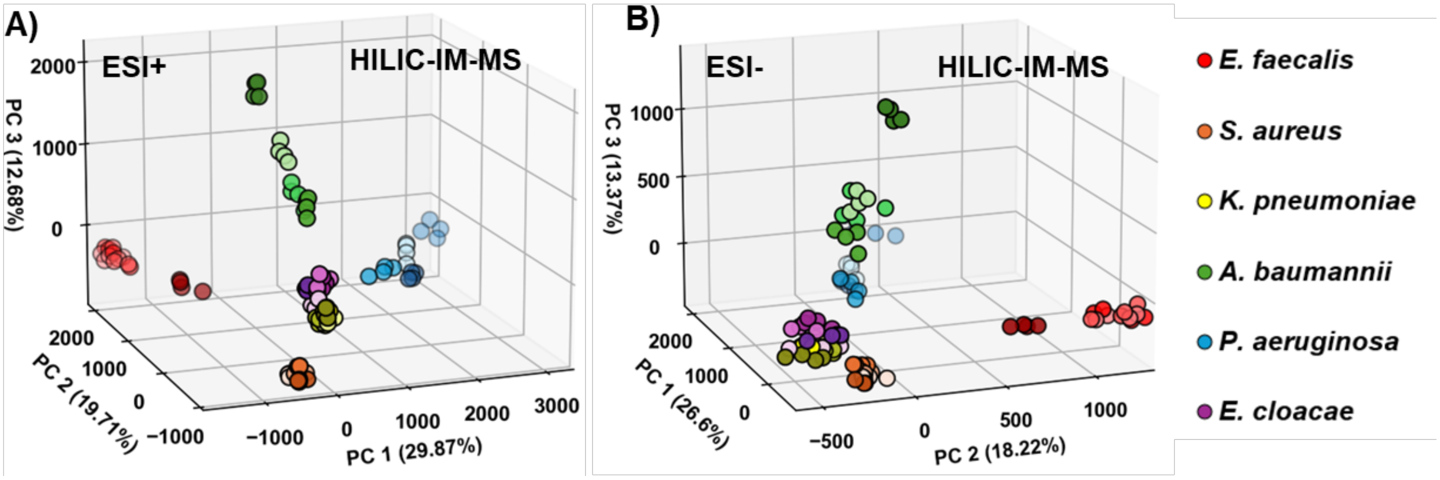

Figure S8. PCA score plots from HILIC-IM-MS measurements of 24 bacteria strains collected in A) positive and B) negative modes. Score plots are based on 10,417 positive mode features and 6,439 negative mode features that were retained after filtering the dataset by ANOVA P≤ 0.000005. Data points are colored with base color of species (6 species) and shade color of strains (4 strains per species, 24 strains).

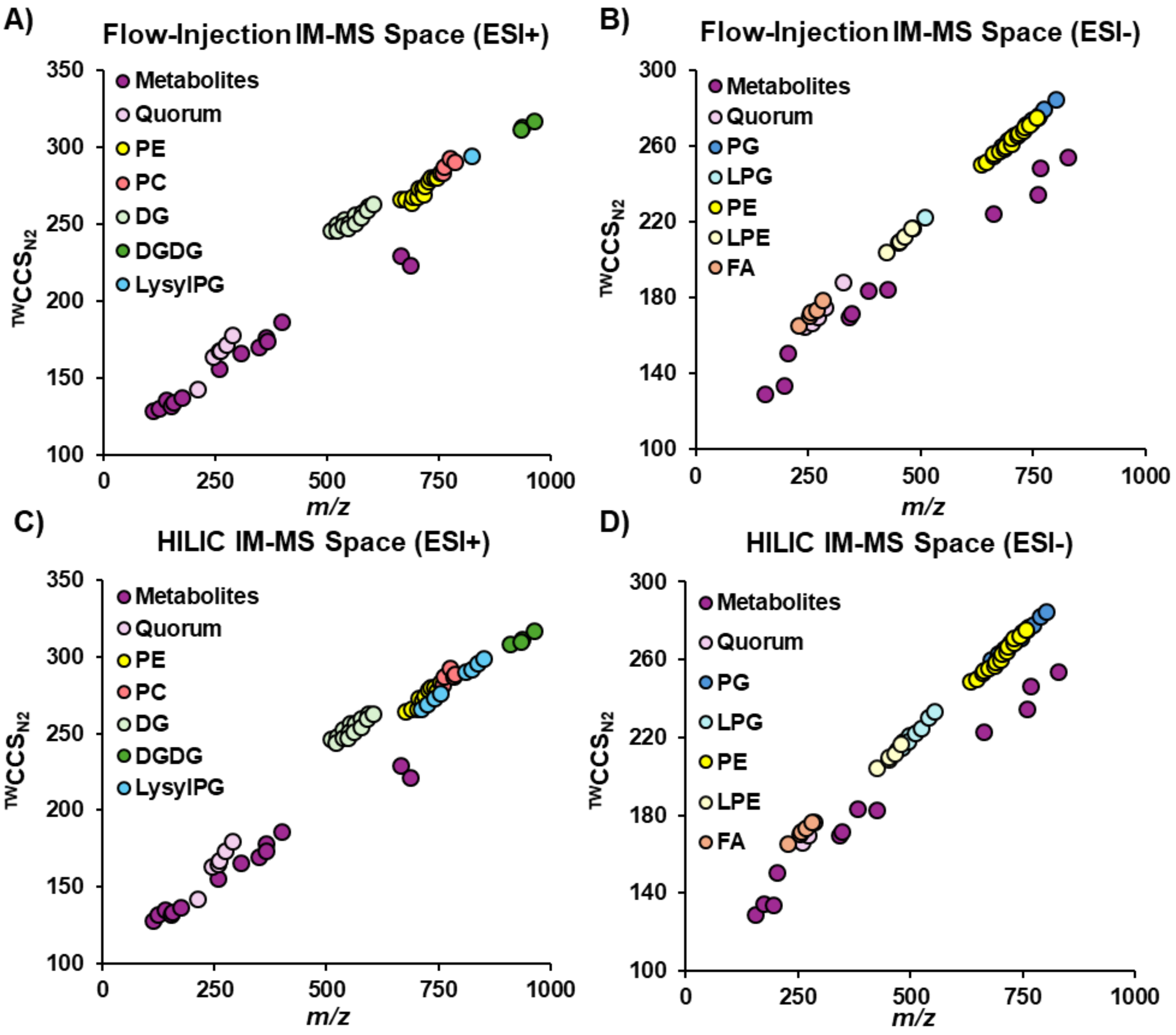

Figure S9. $^{TW}CCS_{N2}$ vs *m/z* plots showing lipids and metabolites detected in the bacterial extracts. Data was collected using flow-injection in A) positive and B) negative ionization mode and HILIC in C) positive and D) negative ionization mode.

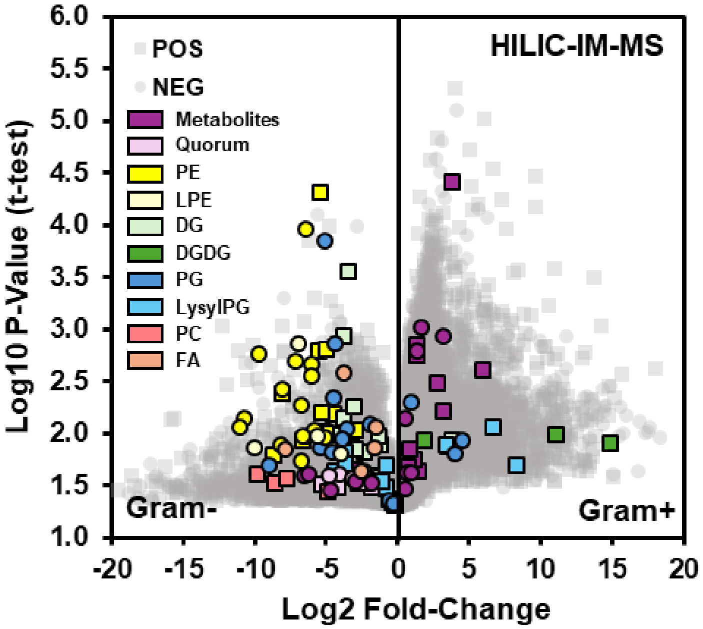

Figure S10. Volcano plot of features found in Gram-Positive and Gram-Negative bacterial extracts analyzed by HILIC-IM-MS in positive (square) and negative (circle) ionization mode. Identified compounds have been colored based on their biomolecular classes.

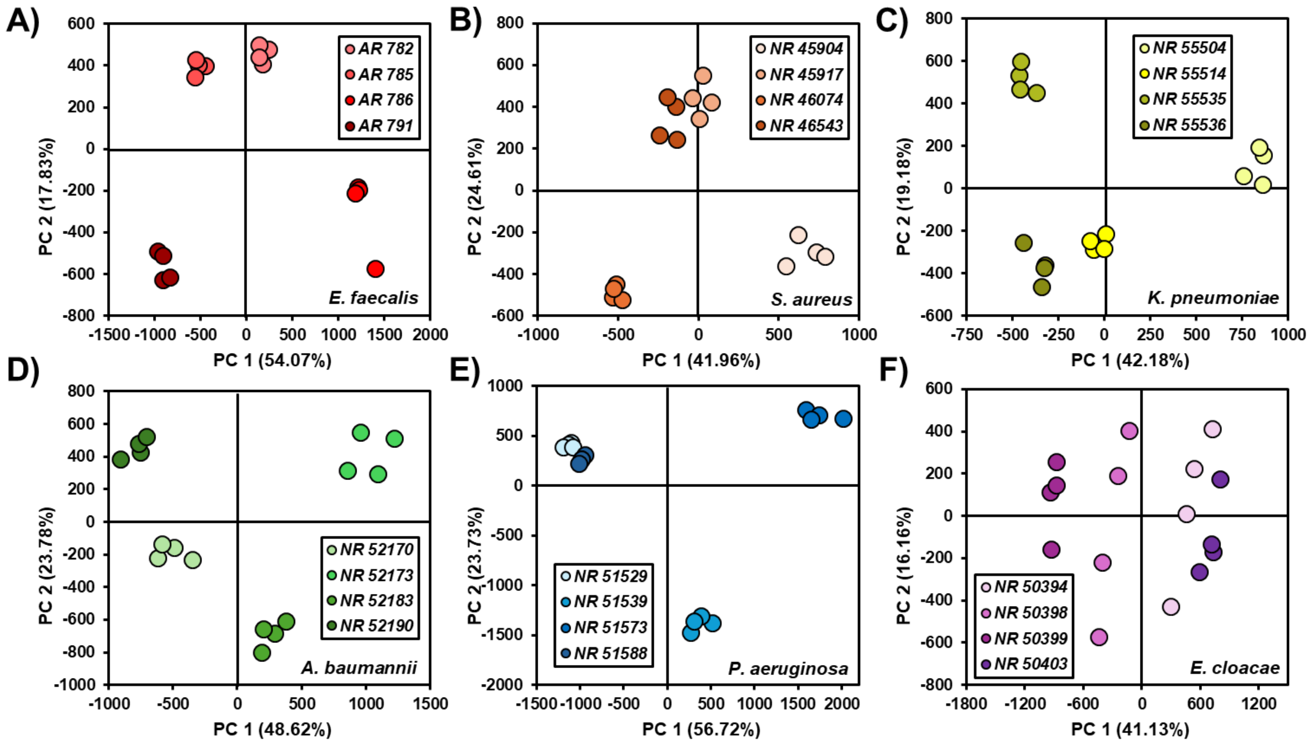

Figure S11. PCA plots of FI-IM-MS measurements in positive ionization mode for 24 bacteria strains and their biological replicates (n=4): A) *E. faecalis*; B) *S. aureus*; C) *K. pneumoniae*; D) *A. baumannii*; E) *P. aeruginosa*; F) *E. cloacae*. Data points are colored by species (base color) and strain (shades of base color).

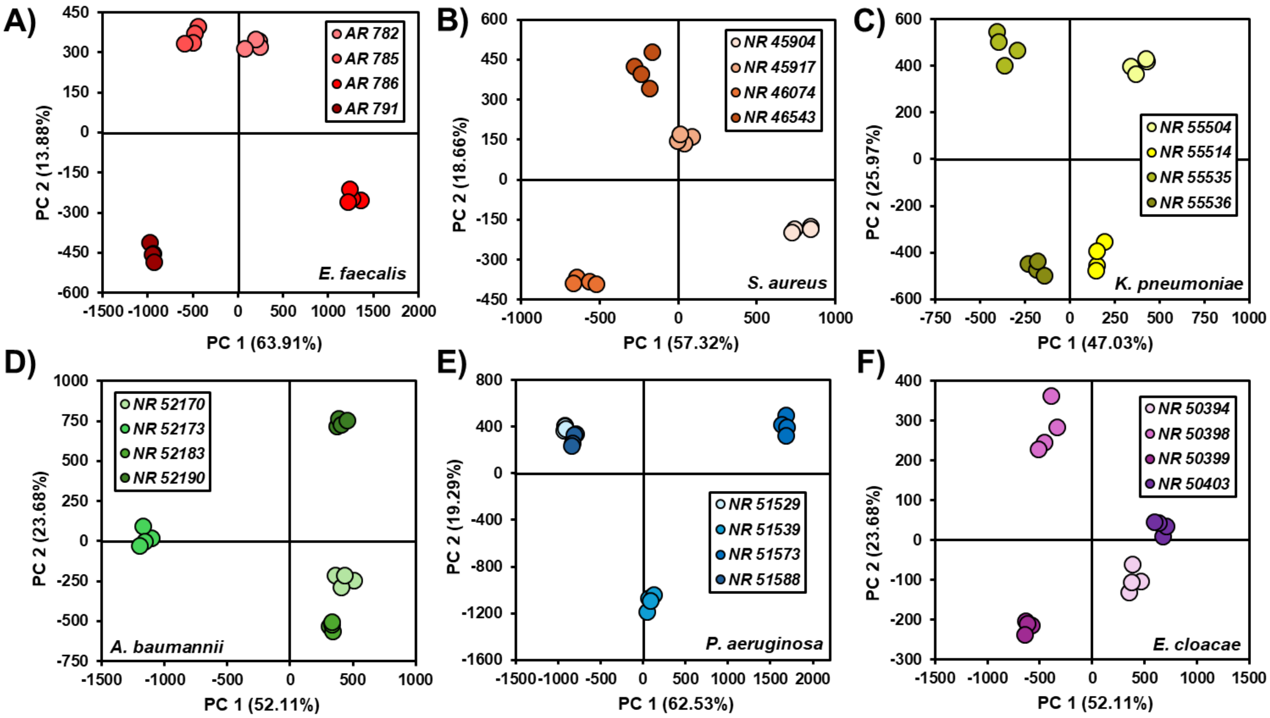

Figure S12. PCA plots of FI-IM-MS measurements in negative ionization mode for 24 bacteria strains and their biological replicates (n=4): A) *E. faecalis*; B) *S. aureus*; C) *K. pneumoniae*; D) *A. baumannii*; E) *P. aeruginosa*; F) *E. cloacae*. Data points are colored by species (base color) and strain (shades of base color).

SUPPORTING INFORMATION REFERENCES

1. Hynds, H. M.; Hines, K. M., MOCCal: A Multiomic CCS Calibrator for Traveling Wave Ion Mobility Mass Spectrometry. *Anal. Chem.* **2024,** *96* (3), 1185-1194.
2. Ding, J.; Ji, J.; Rabow, Z.; Shen, T.; Folz, J.; Brydges, C. R.; Fan, S.; Lu, X.; Mehta, S.; Showalter, M. R.; Zhang, Y.; Araiza, R.; Bower, L. R.; Lloyd, K. C. K.; Fiehn, O., A metabolome atlas of the aging mouse brain. *Nat. Commun.* **2021,** *12* (1).
3. Ulmer, C. Z.; Koelmel, J. P.; Ragland, J. M.; Garrett, T. J.; Bowden, J. A., LipidPioneer : A Comprehensive User-Generated Exact Mass Template for Lipidomics. *J. Am. Soc. Mass. Spectrom.* **2017,** *28* (3), 562-565.
4. Picache, J. A.; Rose, B. S.; Balinski, A.; Katrina; Sherrod, S. D.; May, J. C.; McLean, J. A., Collision cross section compendium to annotate and predict multi-omic compound identities. *Chem. Sci.* **2019,** *10* (4), 983-993.
5. Ross, D. H.; Cho, J. H.; Xu, L., Breaking Down Structural Diversity for Comprehensive Prediction of Ion-Neutral Collision Cross Sections. *Anal. Chem.* **2020,** *92* (6), 4548-4557.
